# Supplementary material for: Towards exploring current challenges and future opportunities relating to the prehospital triage of patients with traumatic brain injury: a mixed-methods study protocol
Source: BMJ Open. 2023 Mar 7;13(3):e068555. doi: 10.1136/bmjopen-2022-068555 (PMC10008429; doi:10.1136/bmjopen-2022-068555)
Supplement: Supplementary data [file bmjopen-2022-068555supp001.pdf]

Semi-structured interview, topic guide

Date: **Participant ID number:**

Pre-interview

- Privacy notice read.
- Consent form.
- Information sheet.

1. In your opinion, how easy/difficult is it to identify a patient with significant TBI who needs major trauma centre care?

2. Where do you think the challenges are? Do you think the current tool you use is an appropriate tool? Why or why not?
   a. Follow-up question, if required: Research suggests that we might under-triage older patients with suspected TBI. Why do you think this might be, and how do you think we could do better?

3. How could triaging patients with suspected TBI be improved in the future?
   a. Follow-up question, if required: What do you think a good triage tool needs to look like/ what does it need to do?
4. What would the ideal TBI triage tool look like if you could design it?
   a. Follow-up question, if required: Is there anything specific that you should be avoided in a triage tool, for example questions that might be too vague or subjective?

5. Can you see any problems with using paramedics' clinical judgement to inform triage decisions?

Alqurashi N, *et al. BMJ Open* 2024; 13:e068555. doi: 10.1136/bmjopen-2022-068555

Introduce new technologies: biomarkers, near-patient infra-red spectroscopy

6. What do you think about the potential to use these technologies in the prehospital field?
    a. Follow-up question, if required: What are the potential barriers for the implementation point-of-care brain biomarkers/NIRS in the prehospital setting in the UK?

7. What would the ideal TBI triage tool look like if you could design it?
    a. Follow-up question, if required: Is there anything specific that you should be avoided in a triage tool, for example questions that might be too vague or subjective?

8. Can you see any problems with using paramedics' clinical judgement to inform triage decisions?

9. Were there any questions you wished we had asked or anything else you would like to discuss?

Alqurashi N, *et al. BMJ Open* 2024; 13:e068555. doi: 10.1136/bmjopen-2022-068555
